# Governance, policy, and health systems responses to the COVID-19 pandemic in Thailand: a qualitative study

**DOI:** 10.3389/fpubh.2024.1250192

**Published:** 2024-03-22

**Authors:** Titiporn Tuangratananon, Nattadhanai Rajatanavin, Sarayuth Khuntha, Salisa Rittimanomai, Nima Asgari-Jirhandeh, Viroj Tangcharoensathien

**Affiliations:** ^1^International Health Policy Program, Ministry of Public Health, Nonthaburi, Thailand; ^2^Asia Pacific Observatory, WHO South East Asia Regional Office, New Delhi, India

**Keywords:** COVID-19, Thailand, health system, governance, policy response

## Abstract

**Background:**

Since 2020, Thailand has experienced four waves of COVID-19. By 31 January 2022, there were 2.4 million cumulative cases and 22,176 deaths nationwide. This study assessed the governance and policy responses adapted to different sizes of the pandemic outbreaks and other challenges.

**Methods:**

A qualitative study was applied, including literature reviews and in-depth interviews with 17 multi-sectoral actors purposively identified from those who were responsible for pandemic control and vaccine rollout. We applied deductive approaches using health systems building blocks, and inductive approaches using analysis of in-depth interview content, where key content formed sub-themes, and different sub-themes formed the themes of the study.

**Findings:**

Three themes emerged from this study. First, the large scale of COVID-19 infections, especially the Delta strain in 2021, challenged the functioning of the health system’s capacity to respond to cases and maintain essential health services. The Bangkok local government insufficiently performed due to its limited capacity, ineffective multi-sectoral collaboration, and high levels of vulnerability in the population. However, adequate financing, universal health coverage, and health workforce professionalism and commitment were key enabling factors that supported the health system. Second, the population’s vulnerability exacerbated infection spread, and protracted political conflicts and political interference resulted in the politicization of pandemic control measures and vaccine roll-out; all were key barriers to effective pandemic control. Third, various innovations and adaptive capacities minimized the supply-side gaps, while social capital and civil society engagement boosted community resilience.

**Conclusion:**

This study identifies key governance gaps including in public communication, managing infodemics, and inadequate coordination with Bangkok local government, and between public and private sectors on pandemic control and health service provisions. The Bangkok government had limited capacity in light of high levels of population vulnerability. These gaps were widened by political conflicts and interference. Key strengths are universal health coverage with full funding support, and health workforce commitment, innovations, and capacity to adapt interventions to the unfolding emergency. Existing social capital and civil society action increases community resilience and minimizes negative impacts on the population.

## Background

As of February 2024, Thailand reported 4.76 million COVID-19 cases and 34,555 deaths; equivalent to 68,006 cases and 493 deaths per million population (1). Thailand ranks 143rd and 139th globally in terms of cases and deaths per million population (2).

In March 2020, Thailand first wave of COVID-19 was triggered by clusters from events at the boxing stadium and night clubs in Bangkok, which later spread to 68 provinces. Harsh responses include total lock down for the whole month of April 2020. Public health and social measures were introduced. These include case identification, quarantine and isolation, contact tracing, and treatment of the patients. After May 2020, evidence reported no local transmissions; all subsequent cases were identified from international travelers in state quarantine sites.

The second wave was triggered by multiple clusters, for example Thai workers returned from work in neighboring countries, carried back corona virus and spread. This cluster spread to several Northern provinces. Further, a large cluster of infection among undocumented migrant workers in a large wholesales seafood market in central province had spread almost all provinces. Similar public health and social measures were applied. This wave subsided in March 2021.

The third wave in April 2021 was triggered by clusters at entertainment facilities, bars and night clubs in Bangkok and spread nationwide during the Thai New Year holidays when large number of people traveled to their provincial hometowns and carried virus. Similar public health and social measures were applied.

The fourth wave was triggered by the delta variant, with 60% higher infectivity than the alpha variant which resulted in significant surge in daily cases and deaths and almost overwhelmed the health systems. The surge of cases made it impossible to conduct contract tracing, strategies focuses mitigate impacts rather than containment. Government introduced triage of patients: mild cases were either put in home or community isolation sites, moderate cases in field hospitals, some equipped with Oxygen facilities, and severe cases in hospital based intensive care facilities.

The fifth wave was caused by the omicron variant, starting in December 2021 with more cases but a lower mortality rate than the delta variant. Similar public health and social measures were applied. By the last quarter of 2022, there was a significant reduction in COVID-19 cases, and high levels of vaccine coverage. The government declared COVID-19 an “endemic” disease and resumed economic activity and initiated a plan for health system recovery.

In 2021, Thailand ranked fifth out of 195 countries and territories for the Global Health Security Index (GHSI), with an index score of 68.5 after United States, Australia, Finland, and Canada. Evidence shows that higher GHSI scores do not consistently predict better control outcome (3). Though leadership and governance determine containment outcomes, it was not included in the GHSI (4, 5). Governance, finance, cross sectoral collaboration, and community engagement are not elements in the GHSI (6).

## Methods

This study applied an inductive qualitative approach with thematic analysis including document review and semi-structured in-depth interviews with policy makers and relevant stakeholders. Our inductive approach applied six health systems building blocks, which include health service delivery, health financing, health workforce, health information systems, medical supplies and leadership, and governance; all of which support effective response to pandemic. We explore how each component contributes to pandemic control as well as policy adaptation and innovations.

### Data collection

We reviewed public and unpublished documents relating to COVID-19 responses including meeting minutes, and government announcements, obtained from the EOC of the MOPH by two of the authors (NR and TT) since 15 May 2020. Participants who were key informants recruited in this study were policy makers who were fully involved in COVID-19 responses from MOPH Departments and related agencies. The identification of key informants for in-depth interviews was guided by our comprehensive review of relevant documents. They key informants had involved in the governance and mobilization of health systems and other social resources to respond to pandemic. We selected them to represent organizations in their respective responsibilities in response to pandemic. We can mobilize only one key informant from Bangkok, which may have resulted in an inadequate number of key informant representing Bangkok compared to the Ministry of Public Health and other organizations (see Table 1).

**Table 1:** Summary of key informants’ affiliation.

Key informant’s affiliation	Major responsibility related to pandemic	Their role related to the six health systems building blocks
Comptroller General’s Department	Managing civil servant medical benefit scheme	Health financing
Department of Medical Science, MOPH	Certify public and private RT-PCR lab, provide reference lab service and monitor variant of concern	Medical supplies
National Health Security Office	Managing universal coverage scheme	Health financing
Government Big Data Institute	Established in 2019 to promote the analysis and management of big data for state agencies	Health information systems
Provincial Public Health Office, MOPH	Implementing pandemic control in the province as secretariat of the provincial communicable disease control committee chaired by Provincial Governor	Leadership and governance
Department of Health Service Support, MOPH	Regulating and mobilizing private clinics and hospitals to provide clinical services to COVID patients	Health service delivery
Department of Medical Services, MOPH	Develop clinical and treatment guidelines and protocol, coordinate all providers in Bangkok	Health service delivery
Food and Drug Administration, MOPH	National regulatory authority, registration of COVID-19 vaccine, and monitor Adverse Event Followed Immunization	Medical supplies
Office of Permanent Secretary, MOPH	Steer overall pandemic control, permanent secretary chairs the MOPH EOC, coordinate with the CCSA	Health workforce, Leadership, and governance
Senior health advisor	Advice the Prime Minister and Minister of Public Health	Leadership and governance
Inspector General Office, MOPH^*^	Oversight, supervise and support the function of provincial health offices	Leadership and governance
National Vaccine Institute	As mandated by National Vaccine Security Act 2018, NVI is an in independent public agency responsible for national vaccine security, funding for national vaccine R&D program, and negotiated with manufacturers of COVID-19 vaccine as well as the COVAX facility	Medical supplies
Thai Public Broadcasting Service	A fully public funded TV broadcaster aiming to provide innovative and comprehensive broadcasting services of a high standard based on code of ethics, public interest, and cost-effectiveness. It was involved heavily in the COVID-19 responses in the role of public media	Health information systems
Department of Disease Control, MOPH	Produce case definition, guidelines on public health response, trace, and contact tracing, support Provincial Health Office on public health measures	Health service delivery
Bangkok Metropolitan Administration (Medical Service Department)	Managing pandemic control in Bangkok through multi-sectoral coordination, providing medical services via its hospitals	Health service delivery
Bangkok Metropolitan Administration (Health Department)	Managing pandemic control in Bangkok through multi-sectoral coordination, providing public health measures through its health centers	Health service delivery
Total	17 participants	

^*^indicates two participants were interviewed.

In-depth semi-structured interviews took place between July and December 2021, each online interview which took between 60 and 90 min. Due to online interview, written consent is not possible, therefore we received verbal informed consent and approval for recordings prior to interview.

The interviewers were trained on qualitative study methods and conducted the interviews with a research assistant. The electronic records were verbatim transcribed in their entirety. Essential components and pertinent issues inherent in the transcripts were systematically categorized into distinct sub-themes. Subsequently, different but similar sub-themes were analyzed and combined to construct overarching thematic domains. These themes emerged from the analysis were discussed and agreed in consensus among all authors to ensure rigor of the analysis process.

### Data analysis and synthesis

Qualitative data obtained from literature and in-depth interviews was analyzed using two approaches: deductive by coding key words from verbatim text in line with the six health system building blocks; and inductive by using thematic analysis to generate new sub-themes and themes or other outstanding unforeseen findings through coding and categorizing contents. In the inductive approach, we used a thematic approach that allows the emergence of salient issues raised by key informants, as well as allowing for patterns or sub-themes to emerge naturally in the conversation. Data retrieved from document review and verbatim text from in-depth interviews was read and organized using Microsoft Excel (2020).

This process involved the systematic extraction of information in the verbatim transcripts into distinct codes, which were generated through a process of open coding, where the data were scrutinized for significant concepts and patterns. These codes were then further refined and grouped into cohesive sub-themes that encapsulated related ideas and concepts. Subsequently, different but similar sub-themes were thoughtfully combined to construct overarching themes, which represented the overall concepts and insights derived from the data. Interview data were triangulated with evidence from literature where relevant. The translation from Thai to English was performed by one researcher and double checked by another researcher (NR and TT) to ensure the same meaning of the interview in Thai language. The findings in each theme had included evidence from literature review and in-depth interview of key informants in a comprehensive manner.

### Ethical consideration

This study was approved by the Institute for the Development of Human Research Protections (number IHRP 742/2564) and the Bangkok Metropolitan Administration Ethics Committee (number E011q/64_EXP).

## Results

Three themes emerged from our analysis, according to interviews from 17 key informants (29.4% Female). Firstly, capacity of the health system and gaps in managing the pandemic; secondly, underlying? population vulnerabilities and inequity; and thirdly, innovative solutions and adaptation.

### Theme 1: managing the pandemic: health systems capacity and gaps

Thailand is widely heralded as a success story with its implementation of Universal Health Coverage, and this continued to be considered true during the pandemic. The first theme, emerged from the interviews, exemplified the contributions and gaps of the six components of health systems to pandemic control.

#### Governance: successes and challenges

The prime minister chairs the CCSA, which decides national policy. While implementation is the responsibility of the Provincial Governor who chairs the multi-sectoral Provincial Communicable Disease Committee, with the Provincial Chief Medical Officer serving as secretary. This mechanism empowers governors to take action in line with the provincial context and resources.

Pandemic control was challenging in Bangkok, as it was the epicenter of waves 3 and 4. Inadequate public health capacity was complicated by complex inter-sectoral coordination across multiple government and private sectors. Of 317 private hospitals nationwide, 94 (30%) are located in Bangkok, and almost all are for-profit enterprises. The MOPH does not have direct command over the BMA. The CCSA recognized there were more problems than solutions in managing the epidemic in Bangkok.

“We (MOPH) can’t do much on pandemic containment in Bangkok; the best we can do is provide technical support rather than direct containment”—K10.

#### Public communication: a major weakness complicated by political conflicts

Public communication failed when several governmental organizations conveyed contradictory and confusing messages to the public. The government’s spokesperson was replaced with healthcare professionals. A few university professors promoted exaggerated messages on Facebook, creating fear about the adverse effects of vaccines and confusing the public (7). The media spread messages about the suffering of households during the peak of daily mortality in August 2021.

“You can't blame people for not understanding the inconsistent announcements (by CCSA)”—K15.

Disinformation about fatal events, encouraged vaccine hesitancy, while government counter-measures were inadequate. Political conflicts also undermined vaccine rollout efforts. Statements in Parliament devalued the vaccine donated by and procured from China, despite WHO Emergency Use Listing (8) and one of the opposition leaders visited the United States asking for mRNA vaccine donations (9). Untruthful social discourse confused the public, i.e., Thai FDA is the main bottleneck of vaccine approval. However, commentators did not understand the vaccine registration process. Delayed vaccine procurement and consequently, unavailability in Thailand, due to global shortages in the second quarter of 2021 led to unfounded charges of corruption.

“Politicizing COVID-19 vaccines might not happen if the government communicates with the public sincerely with information, though the protracted political conflicts are the root causes”—K15.

#### Managing information systems: more problems than solutions

Lack of harmonization and inter-operability of data systems hampered effective multi-sectoral coordinated responses. Public and private RT-PCR results were not centrally pooled, leading to repetition of tests and delayed treatment.

Vaccine coverage in different population groups was reported through different unsynchronized databases. These challenges were later solved with assistance from the Government Big Data Institute (GBDI). Vaccine booking was chaotic as several organizations developed their own mobile applications and untimely reporting of vaccine coverage.

“Linking health data is not only a technical issue, it’s always related to institutional politics and user behavior”—K4.

#### Provision of service: beyond the limits and adaptation

The provision and reimbursement of RT-PCR was restricted to strict case definition; this caused spread of infection among those who were not detected and isolated. The case definition became flexible and in July 2021, Antigen Test Kits were adopted and allowed flexibility in case detection.

The high numbers of cases in 2021 overwhelmed the health system especially in BMA, disrupting the provisions of non-COVID-19 services. In response, public and private hospital beds under BMA’s control were pooled and managed centrally to maximize efficiencies. Despite this measure, bed shortages remained and field hospitals with oxygen and high flow ventilators were created in Bangkok. However, these facilities were fully occupied from large surge of moderate and severe cases. Therefore, private sector, NGOs, the media, and volunteers stepped in to provide support on a voluntary basis and donation in supporting home and community isolation.

The fully occupied field hospitals pushed the government to finally adopt a home and community isolations policy for cases with mild conditions.

#### Health financing: public financial management challenges

The Government Procurement and Inventory Management Act B.E. 2560 (2017) regulates the procurement of products available on the market. In this Act, there is no provision relating to “advance market purchasing or down payment” of COVID-19 vaccines still under research and development (R&D), which is the only mode of securing vaccines by any country. The Bureau of Budget challenged who would be responsible for the unreturned down payment if R&D failed. To overcome this, the National Vaccine Committee, [of which one of the authors (VT) is a member] endorsed the decision, which interpreted that procurement in public health emergency situations can apply “advance market purchasing.” It was then endorsed by the Cabinet, which triggered the process of bilateral negotiations with vaccine manufacturers and the COVAX facility.

“The procurement regulation should not apply to a pandemic situation. The Government Procurement and Inventory Management Act cannot facilitate advance procurement of vaccines. If politicians play safe and wait to purchase when there are vaccines in the market; all the vaccines will be booked by other countries”—K14

#### Public sector health workforce: professionalisms and commitment

Medical and nursing staff, all well-trained, were mobilized from MOPH provincial hospitals to support field hospitals in Bangkok and other hot-spot provinces on an *ad hoc* basis. Retired professionals and volunteers were mobilized. Extra compensation was provided to all frontline healthcare workers. BMA has limited public health capacity with only one epidemiologist, and although MOPH mobilized epidemiologists to support BMA, the number was far from sufficient. Village health volunteers played a critical role as they monitored outsiders who traveled into the community, supported home and community isolation, advocated target populations for vaccination, and supported monitoring adverse events followed immunization. It should be noted that the ratio of VHVs in Bangkok, is much lower than the country as a whole (see Table 2).

**Table 2:** Demographic and health system information of Thailand and Bangkok.

	Thailand	Bangkok
Land area (10)	510,890 sq.km.	1,568 sq.km.
Population size (11) (2021)	66,183,825	5,574,497
Age over 60 (11) (2021)	12,102,022 (18%)	1,160,945 (21%)
Sex ratio (M:F) (11) (2021)	0.95:1	0.88:1
Literacy rates (11) (2019)	93.9	95
Unemployment rates (11) (2021)	2.20%	3.70%
Urban population (11) (2020)	51%	100%
Household income (11) (2021)	27,352 THB	40,200.77 THB
Nature of government	Constitutional monarchy, centralized government	Special administration area headed by elected governor, though supervised by Ministry of Interior
Health service delivery system (12) (2020)	Three-tier health system (primary, secondary, and tertiary care), public MOPH dominated health delivery systems.	Insufficient number of public and private primary healthcare to respond to demand of large population, dominated by super-tertiary hospitals, more private, almost for-profit, than public hospitals: 94 Private hospitals with 13,165 beds while 38 Public hospital with 16,970 beds (MOPH 4,351 beds)
	223 Private hospital and 19,490 beds	
	1,001 Public hospital and 116,689 beds (MOPH 106,206 beds) (Exclude BKK)	
Population bed ratio (11) (2020)	393	183
Population per public primary health care center (12) (2020)	5,737 (Exclude BKK)	34,624
Village Health Volunteer (13) (2020)	1,040,000	15,000
Population per VHV (11, 13)	63.63	371.63

#### Access to essential medical products and technology: lack of global solidarity

Resource scarcity, especially during the first wave in early 2020, occurred when China prohibited export of raw materials to manufacture surgical masks. Consequently, nine factories in Thailand discontinued production. In addition, half of the masks were produced for the export market. This led to introduction of export restrictions by the Ministry of Commerce (14). The situation improved during mid 2020 when China removed export restrictions. In 2021, the Government Pharmaceutical Organization ramped up local production of Favipiravir raw materials and finished products (15) licensed by Thai FDA (16).

While all ASEAN member states except Thailand participated in COVAX, Thailand continued to discuss with COVAX, and at the same time was in parallel bilateral negotiations with potential manufacturers. Advance market purchasing was signed with AstraZeneca (AZ) with a Thai manufacturer (Siam Biosciences) acting as vaccine supplier. In the second wave, no vaccine was available except Sinovac donated and purchased from China, while in the third and fourth quarter of 2021, AZ vaccines became available.

“Vaccine scarcity revealed that every country only cares for themselves, us too. There was no such thing as global solidarity. At the end, countries competed for as many vaccines as they could find”—K14.

### Theme 2: underlying vulnerabilities and inequity

This theme focuses on factors beyond the six building blocks of health systems, which hampered the efforts of pandemic containment. Vulnerability in the community, poverty, living in congested urban dwellings, lack of access to health services, and inadequate community resilience makes communities prone to high levels of infection and mortality. Protracted political conflicts also further complicated effective government interventions.

#### Migrant workers: policy responses to the most vulnerable group

The second wave emerged when traffickers smuggled migrants across the porous border between Thailand and its neighbors. These undocumented migrants were not tested and caused virus spread in their community (17).

The Cabinet decided that all COVID-19-related services, including those used by the documented migrants, should be paid by the government, with the NHSO covering the cost for Thai citizens and the Department of Diseases Control managing payments for all non-Thai people. To facilitate undocumented migrants’ access to care, the government offered opportunities to register with the Labor Ministry before 13 February 2021, allowing migrants to remain legally in the country for another 2 years and have access to COVID services (18).

Populations from lower socioeconomic status such as those living in congested communities and slum areas, crowded households, and migrant workers were affected most by the pandemic as they were exposed to infections when earning a living and could not work from home. 26% of slum residents lost their jobs and 10% relied on donated food, the government’s rescue programs did not reach them effectively (19). Digital divides are main barriers for the vulnerable population who do not have internet literacy, such as electronic registration via mobile application, requirement of a bank account for electronic bank transfer.

“The poor households hardly find the next meals for the family; if we wait until we have zero COVID-19 to re-open the economy, this country may starve and the economy may stagnate”—K11.

Unlike in factories where an outbreak can be contained, the strategy of “localized lockdown, treatment, and isolation” of certain major areas of infection is impossible in BKK, especially in urban slums where there are no clear geographical boundaries. Further, the lockdowns faced negative reactions and resistance from local citizens who had to earn their daily living to survive.

#### Protracted political conflicts and mistrust

Trust in government institutions ensures population adherence to public health and social measures. False criticisms in social media undermining the government were not rebutted by effective communication. Political conflicts created misinformation about vaccines (20, 21). A respected senior vaccine expert was falsely charged with advocating certain vaccines and being “a salesman” of inactivated vaccine. ATK, purchased by the Government Pharmaceutical Organization was accused of being of substandard quality (in terms of sensitivity and specificity) and high cost, however the subsequent anti-corruption investigation reported no evidence of corruption in the procurement procedures (22).

“Any Issue in Thailand can be linked with politics (due to unresolved political conflicts), regardless of its reality”—K11.

Private hospitals wanted to play a role and make profits from the COVID-19 vaccine when “alternative vaccines” (Moderna and Sinopharm) would be delivered by private hospitals at cost. While public vaccines (Sinovac, AstraZeneca, and Pfizer) are free. Social media deliberately devalued the public vaccines as being of low efficacy while advocating Moderna to stimulate the demand (23). The private hospitals’ criticism that the government prohibits them from directly importing vaccine, while in reality, vaccine manufactures could only sell to governments and not private entities under the WHO Emergency Use Authorization.

#### Political interference delays effective responses

Political interference occurred at all levels. NHSO struggled with the procurement of ATK as, unfounded, it was accused of non-transparency on price and the technical specification, despite that the products were registered by the Thai Food and Drug Administration.

Medical experts who reviewed and issued clinical practice guidelines also faced political pressures for inclusion of certain products in the guidelines. Entertainment and hotel sectors put strong pressure on the Government to open their pubs, bars, and Karaoke despite the surge in cases. There was constant pressure, dilemmas, and political choices to be made between business and the economy and the health of the population, and decisions required evidence and building consensus.

### Theme 3: innovative solutions and adaptation

This theme emerged as key informants highlighted various adaptive responses as the pandemic unfolded, especially relating to the size of infectious cases and mortality rates, and resources available through various innovations.

#### Health service delivery systems: technological innovation

Pool saliva tests reduced RT-PCR costs by five times as it did not affect the sensitivity with specimens that PCR cycle threshold was lower than 35 (24).

Innovative heterologous vaccination schemes were introduced prior to the confirmation by the WHO EUL COVID-19 vaccine. Five out of the 48 global references used by the WHO Strategic Advisory Group of Experts on Immunization (SAGE) in the interim recommendations on heterologous vaccine schedule are studies from Thailand (25).

A “Bubble and Seal” strategy in factories was introduced to continue manufacturing while preventing community spread; the factory and routes to and from workers’ dormitories in the factory campus were completely sealed. All symptomatic cases were treated inside the factory, while severe cases were referred to hospitals (26). Another service delivery innovations was the home isolation (HI)/community isolation (CI), which shifted patients from hospital to their own home or community with provision of food, thermometer, and pulse oximeter and monitoring using telemedicine, all reimbursed by NHSO. To increase capacity for managing cases with moderate symptoms, hotels rooms could be converted into hospitals called “Hospitel.” The hotel must affiliate with a hospital to provide clinical services. This innovation maximized the use of over 100,000 hotel rooms nationwide.

“We may take a month to establish a field hospital, but we took a week to have 10,000 rooms in ‘hospitel’ (by transforming hotel rooms into a temporary hospital)”—K6.

#### Multi-sectoral collaboration, social capital, civil society, and active citizens

Multisectoral and inter-ministerial collaboration such as the Government Big Data Institute filled the gap of data integration. Police and armed forces monitored quarantine sites and labor trafficking. The private sector manufactured N95, and the Ministry of Commerce regulated the price and supply. The private sector supported service provision, such as pooling beds and ICU facilities, treatment of severe case, RT-PCR tests, and vaccination. Private banks, telecom companies, and technology start-ups also assisted the public sector by providing IT systems for bed management, and patient support for treatment and counseling.

“With help from the private sector for both equipment and health care workforces, we can expand ICU in just a short period of time”—K9.

A community pantry campaign run by individuals, civil society organizations, and temples aimed to ensure food was available for anyone affected by the pandemic. Food stocks were refilled regularly. Food pantries nation-wide reflected the voluntarism and social capital embedded in Thai society (27, 28).

“NGOs were extremely active and always willing to help us throughout this hardship”—K16.

All sub-themes emerged from participants’ views and not from literature review. However, we did not add quotes to every emerging sub-theme. Further, sub-themes 1.4 provision of service: beyond the limits and adaptation, sub-theme 1.6 public sector health workforce: professionalisms and commitment, and sub-theme 2.3 political interference delays effective responses; emerged from the analysis of interview verbatim.

**Figure 1:**
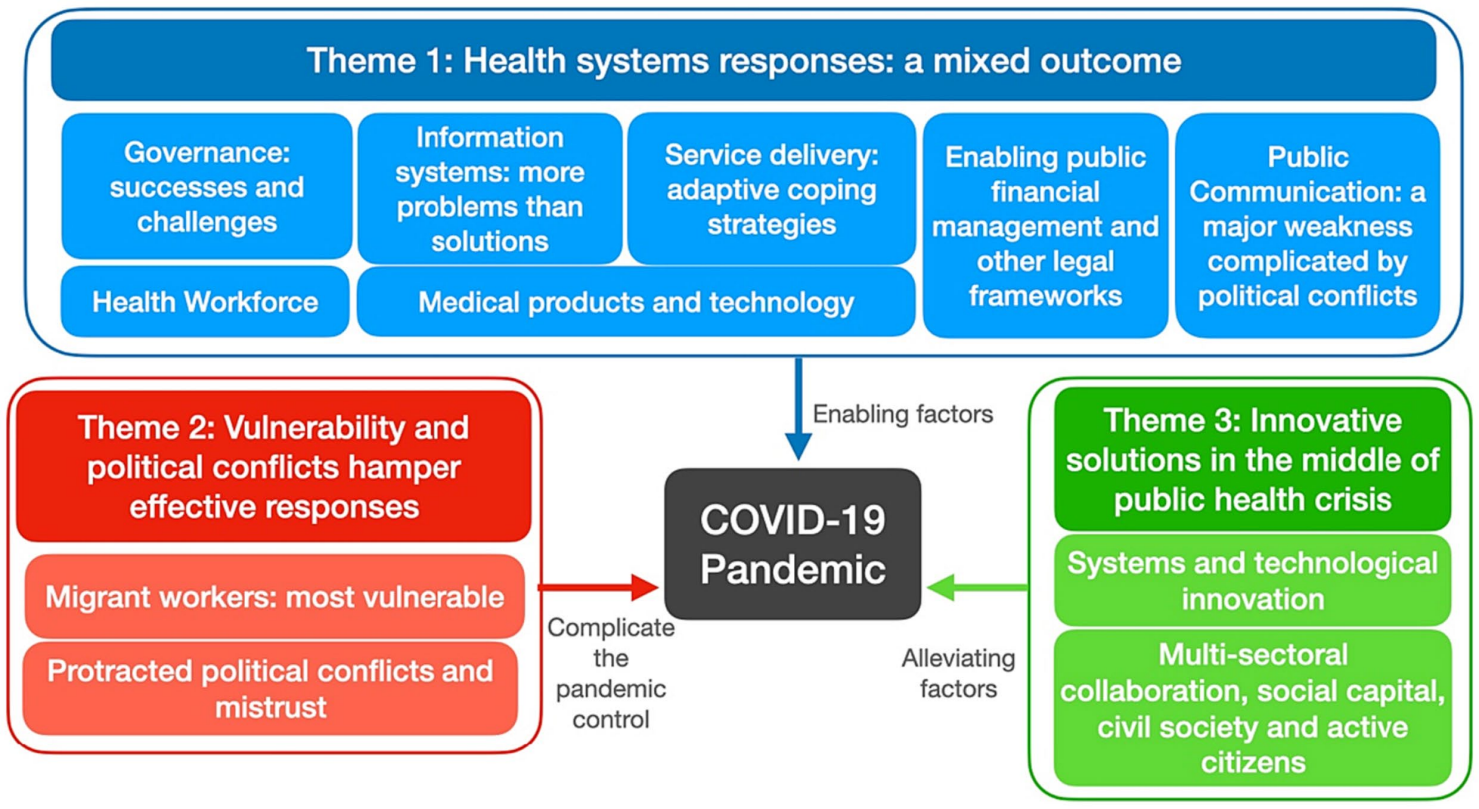
Factors which contributed and hampered effective pandemic control, Thailand.

Figure 1 depicts factors that contributed to and hampered effective pandemic control. The impact on the six building blocks identified in Theme 1 is thoroughly described, taking into account that the Thai health system can provide universal health coverage in a stable situation. Theme 2 is concerned with external factors beyond health that complicated the effective pandemic control such as politics and inequity. Innovative technology and social solutions identified in Theme 3 are mitigating factors, which minimized the direct and indirect consequences of pandemic.

## Discussion

The COVID-19 pandemic seriously affected the health and the economy worldwide. It was described by the World Bank as “double shock,” which refers to both health and economic shocks (29). A modeling study reports 14.9 million global excess mortality over 2020–2021, with a 95% credible interval of 13.3, 16.6 million. The ratio of excess deaths to reported COVID-19 deaths was 2.75, reflecting a huge discrepancy (30). A study showed that all-causes cumulative excess deaths among the Thai population in 2021 was 14.3% (95% CI: 8.6–18.8%), higher than the baseline estimates from the previous 5 years. Excess deaths in males were higher than in females by approximately 26.3%. The excess deaths directly caused by COVID-19 infections accounted for approximately 75.0% of the all-cause excess (31).

These devastating shocks call for an “after-action review” of how Thailand responded to the pandemic, using the lens of governance, policy, and health systems building blocks. This study identifies lessons on how a country should be better prepared and respond to future public health emergencies and pandemics. When the COVID-19 pandemic unfolded, high- and middle-income countries applied different response strategies ranging from elimination to suppression to mitigation (32, 33). Lockdowns must balance the negative impact on the economy and employment (34, 35), as it resulted in negative growth of GDP of minus 6.1% in 2020 (36). These lockdown responses were similar in other ASEAN countries. Singapore and Vietnam adopted lockdown measures in March 2020 with success in curbing infections. Philippines, Indonesia, Laos PDR, and Malaysia also applied lockdown measures, prohibiting public gatherings, international and domestic travel bans, and working from home (37)

Unlike countries above, Thailand adopted “suppression measures” during the December 2020. At that point, number of daily severe cases was within the ICU bed capacity, and the government cautiously continued economic activity (17). Thailand realized that elimination was impossible as border closure was ineffective at stopping illegal immigration via natural land borders (38).

During the third and fourth waves in 2021, suppression strategies through targeted lockdowns could not cope with the rising cases. On 13 August 2021, the pandemic peaked at over 23,000 daily cases and on August 18, daily deaths peaked at 300; this was caused by the Delta variant (39). A mitigation strategy was adopted, with the aim to save lives and extend supply-side capacity. Home and community isolation strategy was adopted for mild and asymptomatic cases, and established large-scale field hospital nationwide. Field hospitals were for moderate cases, which allow hospitals for the treatment of severe cases. In ASEAN countries such as the Philippines, Malaysia, and Vietnam, the surge of Delta cases led to staff shortages, burnout, and insufficient numbers of hospital beds (40). Overwhelming numbers of cases disrupt the provisions of essential health services.

These adaptive responses require adequate governance and health systems capacity, as reflected in Theme 1, and adaptive innovations, as reflected by Theme 3 of this study. Home and community isolation, field hospital beds, use of antigen test kits, and teleconsultation are among key innovations. The adaptive strategies were guided by epidemiological evidence, feedback, and reality checks with frontline implementers as part of the coproduction of evidence (41). Further health systems capacities to deliver public health and medical services, and to support social measures and engage civil society organizations, as reflected in Theme 3, are equally important.

High vulnerability among members residing in congested communities in Bangkok, large numbers of unregistered migrant workers, the lack of capacity of Bangkok local government, and limited coordination with public and private sectors were key barriers. This resulted in Bangkok being an epi-center for COVID-19 and infections spread to other provinces. This issue was raised by the opposition party in the no-confidence debate in September 2021 (42).

This study identified that the main strength, which supported the national response to the pandemic was UHC. It ensured all Thai and non-Thai populations have full access to health services through the three public health insurance schemes, and a significant additional budget approval by the Cabinet allowed for rapid execution (43). However, as Theme 2 uncovered, the underlying vulnerability was found in migrant communities where some are covered by Social Health Insurance and voluntary insurance, but the vast majority are not (44). There was a need to provide services to them in order to contain the pandemic and the government made a quick decision to provide all COVID-19 related services, including vaccines, free of charge for all non-Thai migrants regardless of status. Other ASEAN countries, such as Singapore and Malaysia, also faced an outbreak triggered by migrants living and working in substandard hygienic conditions (45). The COVID-19 pandemic highlighted the underlying challenges in Thailand, especially human and labor trafficking, that require strong political commitment.

For better preparedness and response to a pandemic, this study draws recommendations for countries of any income status. First, strengthening preparedness capacity notably one health surveillance and genomic sequences for risk assessment of pandemics potential pathogens through regional collaboration and support. Sharing of genome sequences through WHO mechanism ensures global health security. Second, at national level, governments need to minimize vulnerability in certain population groups who were affected most by the pandemic, strengthen primary healthcare, progressive realization of universal health coverage; all of which will strengthen health systems resilience. Third, governance during pandemic should be characterized by agility, adaptability, use of sciences, mobilization of whole-of-government and social capitals, and addressing misinformation.

## Limitations

We identified a few limitations. First, the interviews took place during the fourth wave of pandemic, when most key stakeholders were fully occupied in managing the pandemic. As a result, while we successfully interviewed 17 key informants, several others could not participate. Second, we planned to solicit different perspectives from the private health sector, civil society, and general population; however, they declined with similar reasons. Also, we can only mobilize one and two informant from the Bangkok Metropolitan Administration office (one from Department of Medical Service and the other from Department of Health), which may lead to inadequate information related to pandemic response in Bangkok area. Therefore, interpretation of findings should be made carefully. Third, online interviews do not allow researchers to observe their working environment and non-verbal responses. Fourth, authors’ affiliation can lead to potential bias. All authors are affiliated with the Ministry of Public Health, while other participants are also working in various government institutions of the same ministry. Given such limitation, we triangulate their views with documents and analyze in a transparent manner.

## Conclusion

Thailand’s pandemic response strategies were guided by science and evidence, which informed adaptive strategies through a whole-of-government approach. The CCSA activated comprehensive responses early on in the first wave and adapted responses accordingly in the following waves. UHC, along with significant additional budget and support to the health workforce, was instrumental for the provision of pandemic services to all Thai and non-Thai patients. Various challenges on pandemic containment in Bangkok was due to inadequate local government capacity, the size of the vulnerable population, socioeconomic vulnerability among the poor and migrant workers, and the politicizing of the pandemic response, all of which presented significant challenges. Technological solutions, social innovations, and social capital mitigated some of the negative consequences.

## Data availability statement

The raw data supporting the conclusions of this article will be made available by the authors, without undue reservation.

## Author contributions

TT, NR, VT, and NA-J: conceptualization. TT and NR: methodology and data analysis. TT, NR, SK, and SR: validation and writing—original draft preparation. VT and NA-J: validation, writing—review, and supervision. SK and SR: data management. TT: Visualization. All authors contributed to the article and approved the submitted version.
